# Modification of translation factor aIF5A from *Sulfolobus solfataricus*

**DOI:** 10.1007/s00792-018-1037-4

**Published:** 2018-07-25

**Authors:** F. Bassani, A. Romagnoli, T. Cacciamani, A. Amici, D. Benelli, P. Londei, B. Märtens, U. Bläsi, A. La Teana

**Affiliations:** 1 Department of Life and Environmental Sciences, Polytechnic University of Marche, Via Brecce Bianche, 60131 Ancona, Italy; 2 New York-Marche Structural Biology Center (NY-MaSBiC), Polytechnic University of Marche, Via Brecce Bianche, 60131 Ancona, Italy; 3 Department of Clinical Sciences, Section of Biochemistry, Polytechnic University of Marche, Via Ranieri 67, 60131 Ancona, Italy; 4 Department of Cellular Biotechnologies and Haematology, Sapienza University of Rome, Via Regina Elena 324, 00161 Rome, Italy; 5 Department of Microbiology, Immunobiology and Genetics, Max F. Perutz Laboratories, University of Vienna, Vienna Biocenter, Dr. Bohrgasse 9, 1030 Vienna, Austria

**Keywords:** Translation factor aIF5A, Post-translational modification, Hypusination, Deoxyhypusine synthase, *Sulfolobus solfataricus*

## Abstract

Eukaryotic eIF5A and its bacterial orthologue EF-P are translation elongation factors whose task is to rescue ribosomes from stalling during the synthesis of proteins bearing particular sequences such as polyproline stretches. Both proteins are characterized by unique post-translational modifications, hypusination and lysinylation, respectively, which are essential for their function. An orthologue is present in all Archaea but its function is poorly understood. Here, we show that aIF5A of the crenarchaeum *Sulfolobus solfataricus* is hypusinated and forms a stable complex with deoxyhypusine synthase, the first enzyme of the hypusination pathway. The recombinant enzyme is able to modify its substrate in vitro resulting in deoxyhypusinated aIF5A. Moreover, with the aim to identify the enzyme involved in the second modification step, i.e. hypusination, a set of proteins interacting with aIF5A was identified.

**Electronic supplementary material:**

The online version of this article (10.1007/s00792-018-1037-4) contains supplementary material, which is available to authorized users.

## Introduction

The protein synthesis machinery includes highly conserved protein factors. Among these are EF-P in Bacteria and a/eIF5A in Archaea and Eukarya (Benelli et al. 2017). Both, eIF5A and EF-P, were initially identified as translation initiation factors that stimulate the formation of (f)Met-puromycin in vitro (Benne et al. 1978; Glick and Ganoza 1975). This view has recently been challenged and it is now established that their role is in translation elongation and termination rather than in initiation. During the elongation phase, they are needed to promote the synthesis of proteins containing successive residues of proline (PPP or PPG) (Gutierrez et al. 2013; Ude et al. 2013; Doerfel et al. 2013; Schüller et al. 2017). The presence of these sequences causes ribosome stalling and eIF5A/EF-P are required for resumption of elongation (Schmidt et al. 2016; Melnikov et al. 2016). Indispensable for this action is the characteristic and unique post-translation modification: hypusination in eIF5A and β-lysinylation in EF-P, which occurs in both proteins at a conserved lysine residue located in the N-terminal domain (Huter et al. 2017). Hypusination is carried out in two consecutive reactions: a first enzyme, deoxyhypusine synthase (DHS), transfers the aminobutyl moiety of spermidine to the ε-amino group of a specific lysine. In the second reaction the intermediate, deoxyhypusine, is converted into hypusine by deoxyhypusine hydroxylase (DOHH) (Cooper et al. 1983; Park et al. 2010). The β-lysinylation pathway in *E*. *coli* and *Salmonella* sp. occurs in three consecutive steps and starts with the conversion of α-lysine to β-lysine by YjeK, a lysine aminomutase. Next, the lysyl-tRNA synthetase YjeA transfers a β-lysine to the ε-amino group of a specific lysine (Lys34 in *E. coli* EF-P) in an ATP-dependent manner. In the last step, YfcM hydroxylates the lysine residue (reviewed in Rossi et al. 2014).

The archaeal aIF5A and the eukaryal eIF5A proteins contain a basic N-terminal domain in which the site of post-translational modification resides and an acidic OB-fold C-terminal domain. In contrast, the bacterial protein is characterized by three domains, the structure of the first two being superimposable with the eukaryal and archaeal protein (reviewed in Dever et al. 2014). In addition to its role as a translation factor, the eukaryal eIF5A has been implicated into a variety of cellular processes including mRNA decay (Zuk and Jacobson 1998), cell cycle progression (Hanauske-Abel et al. 1994), apoptosis (Caraglia et al. 2003), cell polarity (Chatterjee et al. 2006; Zanelli and Valentini 2005), retroviral infection (Hoque et al. 2009) and stress responses (Gosslau et al. 2009). However, it remains to be clarified whether eIF5A is endowed with different functions or whether these phenotypes result indirectly from its role in translation.

All archaeal genomes sequenced to date contain aIF5A genes. However, some organisms contain the hypusinated version of the protein, while others contain the deoxyhypusinated one, and very few, both versions of the protein (Bartig et al. 1990). Since so far only homologues of the DHS enzyme have been identified but no homologs of the second enzyme, DOHH, the archaeal hypusination pathway remains unsolved. A partial characterization of the protein has been carried out in *S. acidocaldarius* (Bartig et al. 1992) and, more recently, in *H. volcanii* (Prunetti et al. 2016). The latter contains only deoxyhypusinylated aIF5A, whose synthesis differs from the canonical eukaryotic pathway: in the first reaction, the DHS enzyme transfers agmatine to the aIF5A lysine, while in the second reaction the agmatinase enzyme leads to production of deoxyhypusine. Alternatively, as described for *T. vaginalis*, the two modification reactions can be catalyzed by DHS, endowed with bifunctional activity (Quintas-Granados et al. 2016). In any case, modification of that specific lysine seems to be important since at least some Archaea (e.g. *S. acidocaldarius*) are sensitive to the DHS inhibitor, *N*^1^-Guanyl-1,7-diaminoheptane (GC7), which causes a rapid arrest of growth (Jansson et al. 2000).

Here, we report studies on the identification of *S. solfataricus* (Sso) DHS and on its enzymatic activity leading to deoxyhypusination of aIF5A. In addition, attempts were made to reveal the interactome of aIF5A with the aim to identify the function required for its hypusination.

## Materials and methods

### Strains, plasmids and oligonucleotides

All strains, plasmids and oligonucleotides used in this study are listed in Table S1.

### Synthesis and purification of recombinant N-His-aIF5A in *E. coli*

Using genomic DNA of *Sulfolobus solfataricus* P2 (Sso), ORF SSO0970 was PCR-amplified with Phusion High-Fidelity PCR Master Mix (Thermo Fisher Scientific) using the forward (Sso0970_NcoI_F) and reverse (Sso0970_BamHI_R) primers listed in Table S1, which contained NcoI and BamHI cleavage sites. The purified amplification product, cleaved with NcoI and BamHI, was ligated into the corresponding sites of the expression plasmid pETM11 (Table S1). The encoded Sso aIF5A protein was designed to contain six histidine residues at the N-terminus followed by a ten amino acids long peptide linker bearing a Tobacco Etch Virus (TEV) protease cleavage site (ENLYFQ). *E*. *coli* ROSETTA (DE3) (pLysS) cells were transformed with the recombinant plasmid (pETM11-N-His-aIF5A) and grown in LB medium (Bertani 1951) containing appropriate antibiotics (Table S1). After reaching an OD_600_ of 0.7, the synthesis of N-His-aIF5A was induced by addition of IPTG to a final concentration of 0.5 mM. The cells were harvested 3 h later and the recombinant protein was purified by affinity chromatography on Ni–NTA agarose resin (Qiagen) according to the manufacturer’s instructions. The purified protein was subjected to digestion with TEV protease to remove the histidine tag, dialyzed against 50 mM Tris–HCl pH 7.4, 150 mM KCl, 5% glycerol. An aliquot of the recombinant aIF5A was used for the production of polyclonal antibodies in rabbits (Eurogentec, Belgium).

### Construction of plasmids pMJ05-aIF5A and transformation of Sso PH1-16

The construction of plasmids (pMJ05-N-His-aIF5A and pMJ05-aIF5A-C-His) was performed as described by Märtens et al. (2015). ORF SSO0970 was first amplified by PCR, as described above, using primers (aIF5A_N-His_NcoI_F, aIF5A_N-His_EagI_R, aIF5A_C-His_NcoI_F and aIF5A_C-His_EagI_R) listed in Table S1. The amplification product was inserted into plasmid pSVA11 (Table S1) and placed under the transcriptional control of the thermophilic factor 55 alpha subunit constitutive promoter (ptf55α); two versions of the vector were used to abut the corresponding aIF5A protein with a His-tag at the N- and C-terminus, respectively. DNA fragments corresponding to the promoter followed by the coding sequence were then cloned into the shuttle plasmid pMJ05 (Albers et al. 2006) giving rise to pMJ05-N-His-aIF5A and pMJ05-aIF5A-C-His, respectively. Sso PH1-16 (∆pyrEF) was transformed by electroporation (Schleper et al. 1992), recovered for 3 days in uracil medium, and then selected in medium without uracil for additional 3 days. Finally, the cells were plated on Brock’s medium (Brock et al. 1972), supplemented with 0.2% w/v sucrose, 0.2% NZamine, Gelrite (0.7% w/v). Single colonies were inoculated in liquid medium without uracil. Then, total DNA was isolated and the presence of the recombinant plasmid(s) was confirmed using pMJ05 specific primers (pMJ05_F and pMJ05_R) listed in Table S1. As controls, Sso PH1-16 and Sso PH1-16 (pMJ05-ptf55α), harboring the backbone plasmid (mock control), were grown and analyzed in parallel.

### Purification of N-His-aIF5A and aIF5A-C-His from Sso

The Sso strains PH1-16 (pMJ05-N-His-aIF5A) and PH1-16 (pMJ05-aIF5A-C-His) were grown at 75 °C in Brock’s medium (composed of Brock’s salts and supplemented with 0.2% NZamine, 0.2% sucrose, pH 3.0). At an OD_600_ of 0.8, the cells were harvested and lysed by sonication in 20 ml of lysis buffer (50 mM Tris–HCl, pH 7.4, 150 mM NaCl, 15 mM imidazole, 10 mM *β*-mercaptoethanol, 1 mM PMSF), and the lysates were centrifuged at 25,000*g* for 30 min at 4 °C. The clear lysates were incubated overnight at 4 °C with 300 µl of pre-equilibrated Ni–NTA agarose resin (Qiagen). The lysates were transferred to Poly-Prep chromatography columns (Bio-Rad, Hercules, CA, USA) and washed with 30 ml of washing buffer (50 mM Tris–HCl, pH 7.4, 500 mM NaCl, 40 mM imidazole). The proteins were eluted with 1 ml elution buffer (50 mM Tris–HCl, pH 7.4, 150 mM NaCl, 250 mM imidazole). The eluates were dialyzed (50 mM Tris–HCl, pH 7.4, 150 mM KCl, 5% glycerol), concentrated using 3 K Amicon^®^ Ultra-0.5 centrifugal filter devices, and analyzed by SDS-PAGE. Aliquots of the proteins were stored at − 80 °C.

### Western blot analyses

The proteins were separated by SDS-PAGE using standard protocols and transferred onto a 0.2 µm nitrocellulose membrane (GE Healthcare) using a Semi-dry blotting apparatus. Protein transfer was performed at 15 V for 20 min in transfer buffer (25 mM Tris, 192 mM glycine, 0.1% (w/v) SDS, 20% (v/v) methanol). After blocking non-specific binding with 5% nonfat milk, the blots were probed either with anti-aIF5A antiserum (used at a 1:10,000 dilution in Tris buffered saline solution containing 0.05% Tween 20 and 5% nonfat milk) or with the anti-hypusine antibody (Millipore). The primary antibodies were detected by horseradish peroxidase (HRP)-conjugated anti-rabbit IgG (Cell Signaling Technology), employing the enhanced chemiluminescent reagent (SuperSignal West Pico PLUS, Thermo Scientific). The images were visualized with a BioRad ChemiDoc™ MP Imaging system.

### Purification and LC–MSMS analysis of native aIF5A from *Sulfolobus solfataricus* P2

Purification of native *S. solfataricus* aIF5A was performed essentially as described for *S. acidocaldarius* aIF5A (Bartig et al. 1992). The fractions obtained after each chromatographic step were analyzed by Dot-Blot using anti-aIF5A antibodies. The purified native aIF5A was loaded on a Mini-PROTEAN TGX Stain-Free Precast Gel (BioRad), and the protein band was excised and analyzed by LC–MS/MS analysis. The gel band was digested with trypsin as described (Mair et al. 2015). After digestion the peptide solution was desalted on a custom-made C18 stagetip (Rappsilber et al. 2007). Tryptic digests were separated on an Ultimate 3000 RSLC nano-flow chromatography system (Thermo Fisher Scientific), using a pre-column for sample loading (PepMapAcclaim C18, 2 cm × 0.1 mm, 5 μm) and a C18 analytical column (PepMapAcclaim C18, 50 cm × 0.75 mm, 2 μm, Dionex-Thermo-Fisher Scientific), applying a linear gradient from 2 to 35% solvent B (80% acetonitrile, 0.1% formic acid; solvent A 0.1% formic acid) at a flow rate of 230 nl min^−1^ for 60 min. The eluting peptides were analyzed on a Q Exactive HFX Orbitrap mass spectrometer, equipped with a Proxeon nanospray source (Thermo Fisher Scientific). The data-dependent mode survey scans were obtained in a mass range of 375–1500 *m/z* with lock mass on, at a resolution of 60.000 at 200 *m/z* and an AGC target value of 3E6. The 10 most intense ions were selected with an isolation width of 1.6 Da, fragmented in the HCD cell at 27% collision energy, and the spectra were recorded at a target value of 1E5 and a resolution of 30000. Peptides with a charge of +1 were excluded from fragmentation, the peptide match and exclude isotope features were enabled and selected precursors were dynamically excluded from repeated sampling for 15 s. The raw data were processed with MaxQuant software package (version 1.6.0.16, http://www.maxquant.org) (Cox and Mann 2008) by searching against the sequence of aIF5A in the background of the *Sulfolobus solfataricus* uniprot (http://www.uniprot.org) and sequences of common contaminants, with tryptic specificity allowing 2 missed cleavages. Carbamidomethylation was set as fixed modification, oxidation of methionine, N-terminal protein acetylation, hypusine and deoxyhypusine on lysines as variable modifications, all other parameters were set to default. Results were filtered at a protein and peptide false discovery rate of 1%. The protein list was further filtered for a minimum of 2 unique and razor peptides. Peptide hits returned by MaxQuant were manually validated.

### Analysis of recombinant aIF5A-C-His by MS

10 µl (0.5 mg/ml) of aIF5A-C-His, produced in Sso PH1-16, was analyzed by LC-MS in order to assess its intact mass and the eventual presence of post-translational modifications. The recombinant protein N-His-aIF5A produced in *E*. *coli* was analyzed and served as a control. High performance liquid chromatography was performed on a Dionex Ultimate 3000 HPLC (Thermo Fisher Scientific) system configured with the Chromeleon 6.0 software (Thermo Fisher Scientific). The proteins were reduced in 100 mM DTT for 30 min at room temperature and then separated on an Aeris Widepore C4 column (3.6 µm particle size, dimensions 2.1 × 150 mm, Phenomenex) running a 6 min step gradient from 10% up to 70% acetonitrile in 0.1% formic acid. The working temperature was set to 50 °C and the flow rate at 300 µl/min. The LC-system was coupled online to the quadrupole-time of flight-mass spectrometer Synapt G2-Si (Waters), operated via the MassLynx V 4.1 software package, using a Z Spray ESI source (Waters). Mass spectra were acquired in the *m/z* range from 500 to 2000 at a scan rate of 1 s and the mass spectrometer was calibrated with a MS spectrum of [Glu1]-Fibrinopeptide B human (Glu-Fib) solution. The data were analyzed with the MaxEnt algorithm to reconstruct the uncharged average protein mass.

### Synthesis of recombinant aDHS-C-His in *E. coli*

ORF SSO0967 was amplified by PCR using Sso P2 genomic DNA and the oligonucleotides (Sso0967_SphI_F and Sso0967_BamHI_R) listed in Table S1. The gene was inserted into plasmid pQE-70 (Quiagen) to obtain a C-terminal His-tagged version of the protein. The aDHS-C-His protein was purified to homogeneity from *E*. *coli* BL21 (DE3) cells harboring (pQE-70-DHS-C-His) as described above.

### Structural analysis of the recombinant aDHS-C-His and N-His-aIF5A by static light scattering

Analytical size-exclusion chromatography and multiangle laser static light scattering was performed with a Superdex S200 10/300 GL increase column (GE Healthcare) equilibrated with buffer containing 20 mM Tris–HCl, pH 7.5 and 150 mM NaCl. The separation was performed at 25 °C with a flow rate of 0.5 ml/min by HPLC (Agilent Technologies 1260 infinity). The recombinant proteins aDHS-C-His and N-His-aIF5A produced in *E. coli* were pre-incubated at 65 °C for 10 min and 100 µl were injected at a concentration of 1.5 mg/ml. On-line MALLS detection was performed with a mini Dawn Treos detector (Wyatt Technology Corp., Santa Barbara, CA) using a laser emitting at 690 nm and by refractive index measurement using a Shodex RI-101 (Shodex).

### In vitro aDHS-C-His activity

The aDHS-C-His (purified from *E. coli*) activity was assayed in vitro after pre-incubation for 10 min at 65 °C. 200 pmol or 1200 pmol of aDHS-C-His were incubated in a final volume of 30 μl, in the presence of 400 pmol of recombinant N-His-aIF5A purified from *E. coli*, 2 mM spermidine, 2 mM nicotinamide adenine dinucleotide (NAD +), 2 mM MgCl_2_, 50 mM glycine–NaOH buffer, pH 9.4, 150 mM KCl and 1 mM DTT. The reaction mixes were incubated for 2 h at 65 °C. The integrity of the proteins was ensured by SDS-PAGE analysis. An eventual increase in mass of intact (undigested) N-His-aIF5A after the reaction was monitored by LC–MS, performed as described above.

### Identification of aIF5A interactors

N-His-aIF5A and co-purifying proteins were isolated from lysates of Sso PH1-16 (pMJ05-N-His-aIF5A). In addition, a mock purification with a lysate of strain Sso PH1-16 (pMJ05-ptf55α) was performed to account for proteins that bind non-specifically to the matrix. The protein purification was performed as described above, except that the NaCl concentration in the washing buffer was lowered to 300 mM. A 100 μl aliquot of each eluate was denatured in 4 M urea, 50 mM ammonium bicarbonate (ABC), before reducing the disulfide bonds with 10 mM dithiothreitol for 30 min at room temperature. Free thiols were alkylated in the presence of 20 mM iodoacetamide in the dark, and the solution was then diluted with 50 mM ABC to 1 M urea. The proteins were treated overnight at 37 °C with trypsin (Promega, Trypsin Gold) at a ratio of 1:50 of trypsin to protein. The reaction was stopped with trifluoroacetic acid, and the peptides were desalted on C18 Stage tips (Rappsilber et al. 2007). The tryptic digests were separated on an Ultimate 3000 RSLC nano-flow chromatography system (Thermo Fisher Scientific) using a pre-column for sample loading (PepMapAcclaim C18, 2 cm × 0.1 mm, 5 μm, Dionex-Thermo-Fisher) and a C18 analytical column (PepMapAcclaim C18, 50 cm × 0.75 mm, 2 μm, Dionex-Thermo-Fisher), applying a linear gradient from 2% up to 35% acetonitrile in 0.1% formic acid at a flow rate of 230 μl min^−1^ over 120 min. The peptides were analyzed on a Q Exactive HF Orbitrap mass spectrometer (Thermo Fisher Scientific), equipped with a Proxeon nanospray source (Thermo Fisher Scientific), operated in a data-dependent mode. Survey scans were obtained at a mass range of 380–1650 *m/z* with lock mass on, with a resolution of 120.000 at 200 *m/z*. The 10 most intense ions were selected with an isolation width of 2 Da, fragmented in the HCD cell at 27% collision energy, and the spectra were recorded at a resolution of 30000. Peptides with a charge of +1, and higher than 6 were excluded from fragmentation. The peptide match and exclude isotope features were enabled and selected precursors were dynamically excluded from repeated sampling for 30 s. Raw data were processed using the MaxQuant software package (version 1.5.5.1, http://www.maxquant.org) (Cox and Mann 2008) and searched against the Sso Uniprot database (http://www.uniprot.org). The search was performed with full trypsin specificity and a maximum of two missed cleavages. Cysteine carbamidomethylation (CAM) of residues was set as fixed, oxidation of methionine, N-terminal protein acetylation as variable modifications and all other parameters were set to default. The results were filtered at a protein and peptide false discovery rate of 1% and LFQ (label free quantification) was used to quantify proteins relatively between the two samples.

## Results

### Synthesis of recombinant aIF5A in *E. coli* and in Sso

**Fig. 1:**
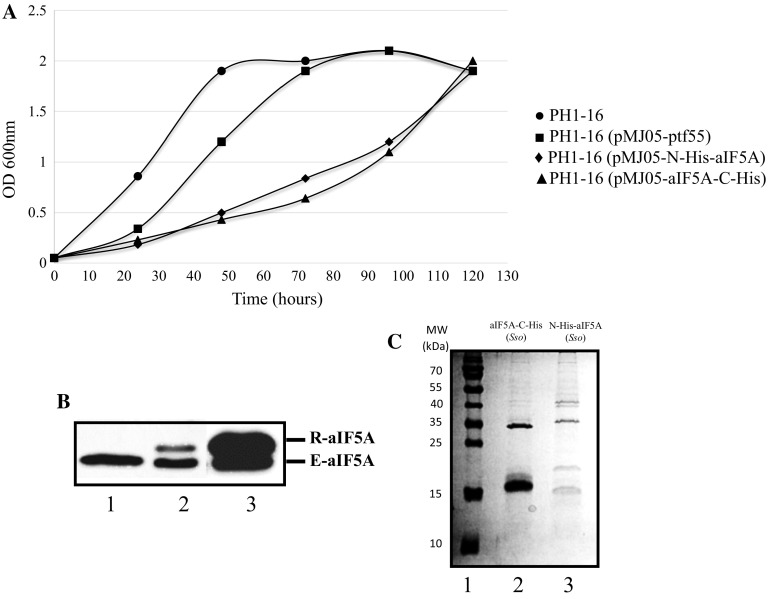
Production of recombinant aIF5A in Sso PH1-16. **a** Growth of Sso strains PH1-16 (filled circle), PH1-16 (pMJ05-ptf55α) (filled square), PH1-16 (pMJ05-N-His-aIF5A) (filled diamond) and PH1-16 (pMJ05-aIF5A-C-His) (filled triangle). **b** Detection of endogenous aIF5A (E) and recombinant N-His-aIF5A and aIF5A-C-His (R) in cell lysates of strains PH1-16 (pMJ05-ptf55α) (lane 1), PH1-16 (pMJ05-N-His-aIF5A) (lane 2) and PH1-16 (pMJ05-aIF5A-C-His) (lane 3), respectively. The cells were grown in Brock’s medium supplemented with 0.2% NZamine, 0.2% sucrose, pH 3.0 to an OD_600_ of 0.8, lysed by sonication, and 100 μg of total protein were subjected to SDS-PAGE followed by western blot analysis with anti-aIF5A. **c** SDS-PAGE of Ni-affinity purified aIF5A-C-His (lane 2) and N-His-aIF5A (lane 3). Lane 1, molecular weight marker (MW). The gel was stained with Coomassie brilliant blue

The gene encoding translation factor aIF5A (ORF SSO0970) was cloned into two different expression vectors to permit the production of the recombinant protein in *E. coli* and Sso. The pETM-11 vector was selected for production of the protein in *E*. *coli* ROSETTA (DE3)/pLysS, allowing the introduction of an N-terminal His-tag followed by a peptide specifically recognized by the TEV protease. Recombinant N-His-aIF5A from *E. coli* was purified to homogeneity as described in Materials and methods, digested with TEV protease and used for production of rabbit polyclonal antibodies. Production of recombinant aIF5A in *S. solfataricus*, was achieved using variants of plasmid pMJ05-ptf55α (Albers et al. 2006), harboring the promoter of the alpha subunit of the chaperonin TF55. Two versions of this plasmid were generated, that permitted the addition of a His-tag at the N-terminal- and at the C-terminal end, respectively (N-His-aIF5A and aIF5A-C-His). Both constructs were used to transform *Sulfolobus solfataricus* PH1-16 (∆*pyrEF*). A control strain bearing the empty plasmid, Sso PH1-16 (pMJ05-ptf55α), was created as well. The two strains containing the recombinant plasmids showed a slow-growth phenotype (Fig. 1a), indicating that the production of the recombinant protein might exert a toxic effect. However, it remains to be studied whether this observation is attributable to increased production of the recombinant proteins or to their activity. The polyclonal anti-aIF5A antibodies detected the N-His-aIF5A and the aIF5A-C-His proteins as well as endogenous aIF5A in lysates of Sso PH1-16 harboring plasmids pMJ05-N-His-aIF5A and pMJ05-aIF5A-C-His, respectively. Likewise, endogenous aIF5A was recognized in lysates of Sso PH1-16 and Sso PH1-16 (pMJ05-ptf55α) (Fig. 1b). Recombinant aIF5A was obtained in high yield and purity from strain Sso PH1-16 (pMJ05-aIF5A-C-His) (Fig. 1c), and used for the following experiments.

### Sso aIF5A is hypusinated

**Fig. 2:**
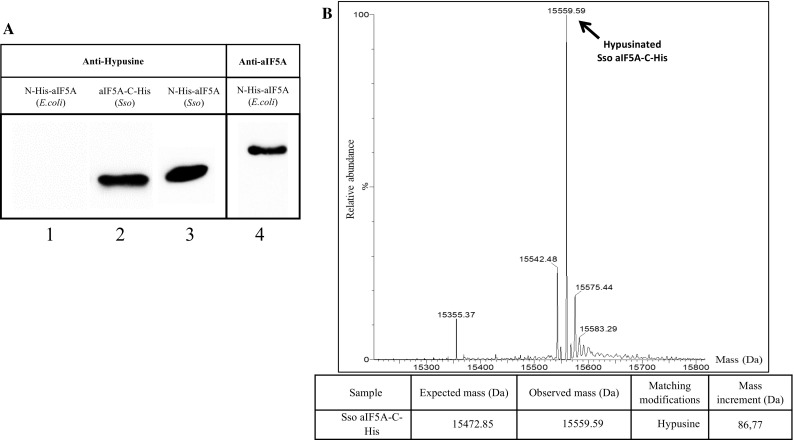
Detection of modified aIF5A. **a** Immunodetection of N-His-aIF5A purified from *E*. *coli* (lane 1), aIF5A-C-His purified from Sso PH1-16 (pMJ05-aIF5A-C-His) (lane 2) and N-His-aIF5A purified from Sso PH1-16 (pMJ05- N-His-aIF5A) (lane 3). The proteins were separated by 15% SDS-PAGE and detected by western blotting using anti-aIF5A and anti-hypusine antibodies. Lane 4, 10 pmol of N-His-aIF5A purified from *E. coli* were separated and probed with polyclonal anti-aIF5A antibodies. **b** MS spectrum of recombinant aIF5A-C-His produced in Sso PH1-16 (pMJ05-aIF5A-C-His). The mass of the main peak (15559.59 Da) corresponds to the anticipated mass of aIF5A-C-His (15472.85 Da) plus the hypusine residue (86.77 Da). The peak with the calculated mass of 15542.48 Da corresponds to the deoxyhypusinated form of aIF5A-C-His

First, commercially available anti-hypusine antibodies were used to test whether Sso aIF5A is modified. These antibodies were raised against a synthetic peptide encompassing the hypusination site of human eIF5A and recognize both, the hypusine or deoxyhypusine modification on eIF5A (Nishiki et al. 2013). As shown in Fig. 2a, the anti-hypusine antibodies specifically recognized the recombinant proteins aIF5A-C-His and N-His-aIF5A (lanes 2 and 3) produced in Sso but not N-His-aIF5A produced in *E. coli* (Fig. 2a, lane 1), which was also probed with the polyclonal anti-aIF5A antibodies mentioned above (Fig. 2a, lane 4). These studies indicated that the protein produced in Sso is modified. To confirm this finding and to clarify the nature of the modification, the mass spectrum of intact aIF5A-C-His from Sso PH1-16 (pMJ05-aIF5A-C-His) was analyzed first. The MS spectrum (Fig. 2b) revealed a main peak, relative to the most abundant polypeptide, with a mass of 15559.97. When compared with the theoretical mass of aIF5A-C-His, the mass increase of 86.77 correlated with the molecular weight of the hypusine residue. A peak corresponding to deoxyhypusinated aIF5A-C-His (15542.48) was also detected, although with a low abundance. Hence, we concluded that most of the recombinant aIF5A-C-His produced in Sso PH1-16 is hypusinated.

**Fig. 3:**
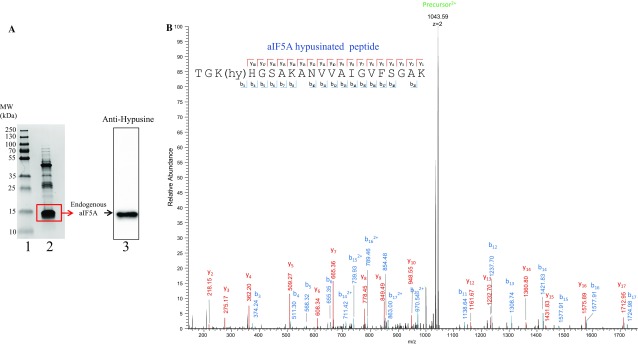
Endogenous Sso aIF5A is hypusinated. **a** Lane 1, molecular weight standards. Lane 2, Coomassie stained endogenous aIF5A purified from *Sulfolobus solfataricus* P2. The rectangle indicates the area that was excised and subjected to LC–MS/MS analysis. Lane 3, western-blot analysis of endogenous aIF5A probed with anti-hypusine antibodies. **b** MS spectrum of the doubly charged precursor 1043.59 Da assigned to the hypusinated peptide TGK(hy)HGSAKANVVAIGVFSGAK. Matching y-ions are marked in red, b-ions in blue

For further verification, we next tested whether the endogenous aIF5A is modified. The endogenous protein was purified (Fig. 3a, lane 2) according to the protocol described for *S. acidocaldarius* aIF5A (Bartig et al. 1992). As the anti-hypusine antibodies recognized endogenous Sso aIF5A (Fig. 3a, lane 3), the corresponding band was excised from the gel, digested with trypsin, and analyzed by LC–MS/MS. Figure 3b shows the spectrum of the hypusinated peptide TGKHGSAKANVVAIGVFSGAK, confirming the hypusine modification at the conserved K36 residue of native aIF5A. The observed mass of the charged precursor was determined with 2087,18 (1043,59 * 2), which corresponds to the mass of the unmodified peptide (1999.30) and the hypusine residue (86.77). It is worth mentioning that no deoxyhypusinated peptides were observed. Peptides covering the unmodified K36 were also detected, however trypsin missed cleavage did not allow an estimation unmodified/modified peptides.

### Identification and purification of Sso aDHS

**Fig. 4:**
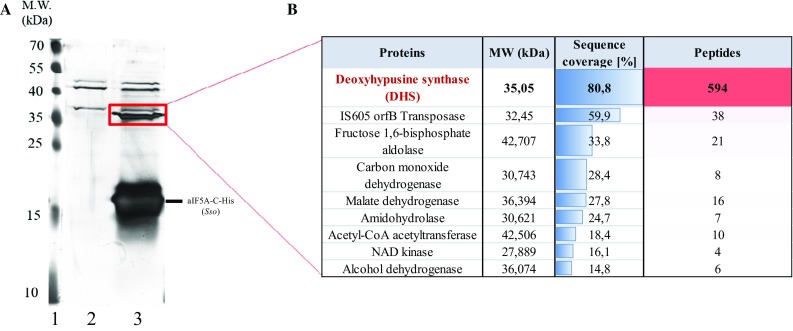
Identification of proteins co-purifying with aIF5A-C-His. **a** Lane 1, molecular weight standards. Lanes 2 and 3, SDS-PAGE of affinity-purified proteins from strains PH1-16 (pMJ05-ptf55α) and PH1-16 (pMJ05-aIF5A-C-His). In-gel visualization of proteins was achieved by silver staining. The rectangle indicates the area that was excised and subjected to LC–MS/MS analysis. **b** The majority of peptides detected in the sample corresponded to deoxyhypusine synthase (aDHS)

During purification of N-His-aIF5A and aIF5A-C-His from Sso PH1-16 lysates, a ~ 35 kDa protein co-purifying with both His-tagged versions of aIF5A was observed (Fig. 4a). As more stringent purification procedures (not shown) did not affect this co-purification, the protein appeared to be tightly associated with aIF5A. The band corresponding to the 35 kDa protein was excised from the gel and subjected to MS analysis (Fig. 4b). The analysis revealed that the indicated band contained predominantly deoxyhypusine synthase (aDHS), the putative first enzyme in the hypusination pathway. Sso aDHS is encoded by ORFSSO0967. The corresponding amino acid sequence was compared to that of the human enzyme using BLAST (https://blast.ncbi.nlm.nih.gov/Blast.cgi). The human DHS has been extensively characterized both, at the structural and biochemical level, and it has been shown that the active form of the enzyme is a homotetramer. The sequence comparison (Supplementary Fig. 1) showed that the two proteins share 59% homology with 36% amino acid identity, and that most of the identical residues comprise those involved in spermidine and NAD + binding, suggesting a common mechanism of action of the two enzymes.

**Fig. 5:**
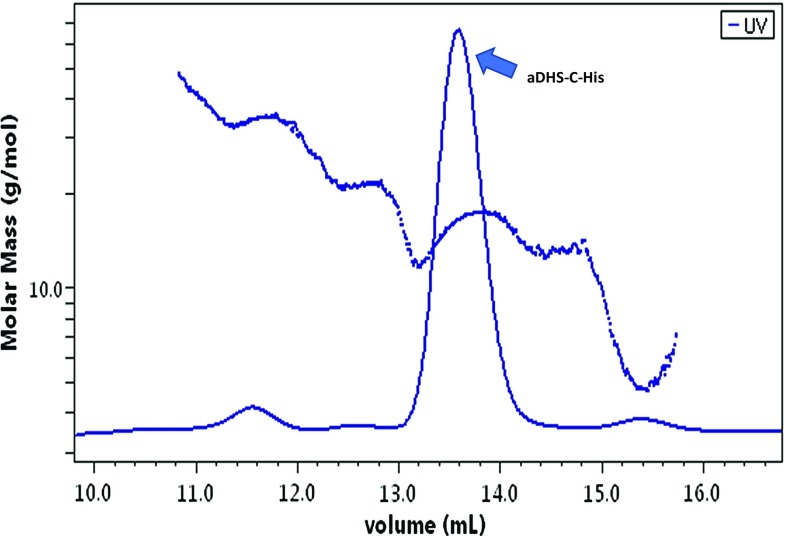
Size exclusion chromatography combined with multi-angle laser light scattering analysis of aDHS-C-His. Elution profile of recombinant aDHS-C-His from a Superdex S200 10/300 GL chromatography column. The absorbance at 280 nm, together with multi-angle light scattering of the sample (dots) were monitored continuously. The single peak (arrow) corresponds to a complex with a molecular weight of 144 kDa

Next, aDHS-C-His was purified from *E. coli* and the oligomeric state was determined by analytical size-exclusion chromatography coupled with multiangle laser static light scattering. The elution profile (Fig. 5) showed a single peak and the light scattering signal revealed a mass of 144.000 Da, corresponding to the tetrameric form of the protein.

### In vitro aDHS assay

The results shown in Figs. 2 and 4 suggested that aDHS is involved in the modification of aIF5A. However, the hypusination pathway remains obscure since no DOHH orthologous have been found in archaeal genomes. In *Trichomonas vaginalis*, hypusination of eIF5A is most likely the result of the catalytic activity of a single bifunctional enzyme (TvDHS), which performs both the DHS and DOHH reactions (Quintas-Granados et al. 2016). Hence, we hypothesized that either Sso aDHS performs the synthesis of only deoxyhypusine, and then a second enzyme, still to be discovered, catalyzes hypusination or, alternatively, aDHS acts like the bifunctional TvDHS catalyzing both modifications.

**Fig. 6:**
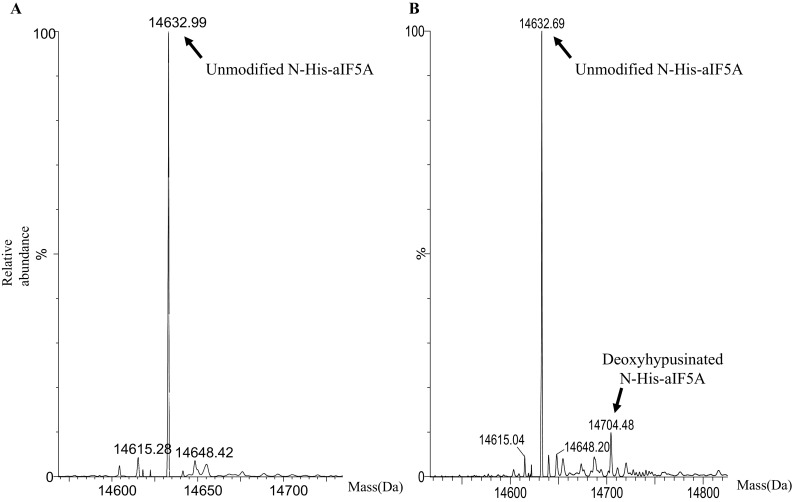
In vitro aDHS-C-His activity assay. N-His-aIF5A purified from *E*. *coli* was incubated together with aDHS-C-His as described in “Materials and methods” **a** MS spectrum of N-His-aIF5A in the absence of the aDHS-C-His (control). **b** MS spectrum of N-His-aIF5A in the presence of aDHS-C-His

To distinguish between these possibilities aDHS-C-His was incubated together with N-His-aIF5A in the presence of spermidine and NAD + as described in Materials and methods. Modification of N-His-aIF5A by aDHS-C-His was assessed determining the protein intact mass by mass spectrometry. Figure 6a shows the obtained spectrum of N-His-aIF5A in the absence of aDHS-C-His. In the presence of aDHS-C-His, a peak was observed, which corresponded to the deoxyhypusinated form of N-His-aIF5A (Fig. 6b). The MW corresponding to the different peaks are listed in Table 1. Several reaction conditions were tested (not shown), nevertheless no peak corresponding to hypusinated N-His-aIF5A was observed. Although the in vitro deoxyhypusination activity of aDHS-C-His was rather low (~ 10% of total N-His-aIF5A), its presence strongly indicates that aDHS is able to perform deoxyhypusine but not hypusine synthesis. We therefore concluded that the second step of the reaction is carried out by an additional enzyme.

**Table 1:** Molecular weights (Da) corresponding to the different peaks of the MS spectra presented in Fig. 6

Sample	Expected mass (Da)	Observed masses (Da)	Matching modifications	Mass increment (Da)
Unmodified N-His-aIF5A	14632.83	14632.99		+ 16
N-His-aIF5A in the presence of aDHS-C-His	Unmodified N-His-aIF5A (14632.83) Deoxyhypusinated N-His-aIF5A (14703.83) Hypusinated N-His-aIF5A (14719.83)	14632.69		− 14
		14704.56	Deoxyhypusine	+ 71

### Identification of aIF5A interacting partners

In a first attempt towards the identification of the second enzyme of the hypusination pathway, proteins co-purifying with recombinant N-His-aIF5A produced in Sso PH1-16 (pMJ05-N-His-aIF5A) were analyzed by mass spectrometry. In parallel, a mock purification from a control Sso PH1-16 (pMJ05-ptf55α) cell lysate was performed to exclude proteins that bind non-specifically to the affinity matrix. Supplementary Fig. 2 shows the pattern of proteins co-purifying with N-His-aIF5A and the proteins present in the mock control. The same samples were also probed with anti-hypusine antibodies to confirm that N-His-aIF5A is modified (Supplementary Fig. 2). The identity of all proteins present in the mock control (Supplementary Figure S2, lane 2) and those co-purifying with aIF5A-N-His (Supplementary Figure S2, lane 3) were analyzed by mass spectrometry. The peptides obtained by mass spectrometry were searched against the Uniprot Sso database. Proteins co-purifying with N-His-aIF5A, which were enriched more than 1.5 fold when compared with the mock control were selected. This analysis identified 31 proteins (Table 2). In agreement with the results shown above, the most abundant protein interacting with N-His-aIF5A was aDHS (122 vs 0 peptides in the mock control), again confirming its tight association with aIF5A. No putative hydroxylase enzymes or proteins with similarity or analogy with DOHH were detected. Nevertheless, several enzymes, such as dehydrogenases and oxido-reductases, with the potential to perform the conversion of deoxyhypusinated to hypusinated-aIF5A were identified, the activity of which can be further analysed.

**Table 2:** Functional categorization of proteins interacting with N-His-aIF5A purified from Sso PH1-16 (pMJ05-N-His-aIF5A), by LC–MS/MS

ORF	MW (kDa)	Experimental peptides	Control peptides	Sso proteins
Cellular metabolism				
SSO0967	35,05	122	0	Deoxyhypusine synthase (aDHS)
SSO0534	42,506	147	63	Acetoacetyl-CoA thiolase
SS01214	23,678	110	0	Carbonic anhydrase
SS02885	35,08	31	0	Oxidoreductase
SSO3050	27,012	26	0	Xylose isomerase
SSO0530	48,508	22	6	Serine hydroxymethyltransferase
SS02779	33,603	21	13	Dehydrogenase
SSO3006	111,16	20	0	Alpha-mannosidase
SS02356	62,531	16	3	Succinate dehydrogenase
SS02219	27,889	14	3	Nad kinase
SS02747	29,71	13	2	Aldose 1-epimerase
SSO0214	34,338	12	0	Acetate kinase
SSO1067	19,227	11	5	Intracellular proteinase
SSO3016	33,011	10	3	Amidohydrolase
SS02514	73,038	9	0	3-Hydroxyacyl-CoA dehydrogenase
SSO0989	48,225	7	0	Sugar phosphate nucleotydyl transferase
SS02521	35,508	7	0	Carboxylesterase
SSO3004	33,096	6	1	3-Oxoacyl reductase
SS01178	20,815	6	1	SAM-dependent methyltransferase
rRNA/tRNA modification and processing, RNA turnover, translation				
SSO0176	85,807	157	74	ATPase AAA
SSO2509	14,786	83	14	Translation recovery factor
SS02226	24,933	82	53	Pseudouridine methyltransferase Nep1
SS02363	43,683	40	1	MoxR-like ATPase
SSO0606	17,975	25	14	Transcriptional regulator, AsnC family
SSO0939	46,692	23	16	Nop56 (C/D box methylation guide RNP)
SS06454	8,679	8	6	SmAP1
SSO0221	12,095	6	1	RPL30e (50S ribosomal protein)
Hypothetical proteins				
SS01412	23,811	50	4	Hypothetical proteins
SS01413	16,072	23	8	Hypothetical proteins
SS01383	20,242	12	0	Hypothetical proteins
SSO0845	27,012	12	1	Hypothetical proteins

The ORFs (ORF) encoding the respective proteins and their molecular weights (MW) are listed in the respective columns. The number of peptides assignable to the identified proteins is shown for the experimental set-up and for the mock control

## Conclusions

In this work, we have shown that Sso aIF5A, like its eukaryal counterpart eIF5A, is hypusinated. In addition, the recombinant protein was found to be tightly associated with deoxyhypusine synthase (aDHS), the first enzyme in the hypusination pathway. Thus, the first step towards hypusination of aIF5A in Sso appears to be similar to that in Eukaryotes. In fact, like the eDHS, recombinant aDHS-C-His enzyme purified from in *E*. *coli* forms a tetramer in solution, which was also the case for aDHS of *Pyrococcus horikoshii* (deposited in the RCSB Protein Data Bank as 4P63). The enzymatic assays with aDHS-C-His resulted in the conversion of only a small amount of N-His-aIF5A into the deoxyhypusinated form. It is formally possible that the enzyme performs a reversible reaction as known in Eukaryotes (Park et al. 2003). Therefore, the hypusination step might be needed to stabilize the modified form of the substrate. In any case, the absence of hypusinated aIF5A from the reaction products seems to exclude the possibility of aDHS being a bifunctional enzyme as described in *T. vaginalis* (Quintas-Granados et al. 2016). The identification of the aIF5A interactome (Table 2) bears the potential to unravel the function(s) involved in hypusination of aIF5A.

## Electronic supplementary material

Below is the link to the electronic supplementary material.

Supplementary material 1 (DOCX 22 kb)

Supplementary material 2 (PPTX 385 kb)

## Abbreviations

aIF5A: Archaeal initiation factor 5A
DHS: Deoxyhypusine synthase
DOHH: Deoxyhypusine hydroxylase
GC7: *N*^1^-Guanyl-1,7-diaminoheptane
