# Supplementary material for: Modification of translation factor aIF5A from Sulfolobus solfataricus
Source: Extremophiles. 2018 Jul 25;22(5):769–80. doi: 10.1007/s00792-018-1037-4 (PMC6105217; doi:10.1007/s00792-018-1037-4)
Supplement: Supplementary file 1 — Supplementary material 1 (DOCX 22 kb) [file 792_2018_1037_MOESM1_ESM.docx]

**SUPPLEMENTARY MATERIAL**

Modification of translation factor aIF5A from *Sulfolobus solfataricus*

F. Bassani, A. Romagnoli, T. Cacciamani, D. Benelli, P. Londei, B. Märtens, U. Bläsi and A. La Teana

**Figure legends**

Supplementary Table 1. Oligonucleotides, plasmids and strains used in this work.

Supplementary Figure 1. Sequence conservation in DHS amino acid residues involved in NAD^+^ and spermidine binding. Pairwise protein sequence alignment of DHS from *Homo sapiens* (gi:1113109) and from Sso P2 (gi:13814149) using BLAST (https://blast.ncbi.nlm.nih.gov/Blast.cgi). Residues, which are in contact with the NAD^+^ cofactor are marked in green, whereas those involved in spermidine binding are marked in red.

Supplementary Figure 2. Purification of N-His-aIF5A from Sso. Lane 1, molecular weight marker (M.W.). (**A**), proteins retained on the matrix obtained from strains PH1-16 (pMJ05-ptf55α) (lane 2) and PH1-16 (pMJ05-N-His-aIF5A) (lane 3) were subjected to SDS-PAGE and visualized by silver staining. (**B**), the section of the gel containing N-His-aIF5A (see lane 3 in panel A) was probed with anti-hypusine antibodies by western-blotting (**B**). Lanes 1 and 2 correspond to lanes 2 and 3 in panel A, respectively.

**Supplementary Table 1**

| **Oligonucleotide** | **Sequence (5’-3’)** |
| --- | --- |
| Sso0970_NcoI_F | GCAACCATGGGCATAACGTACACG |
| Sso0970_BamHI_R | GCGCGGATCCCTTAACCCTAACTATT |
| Sso0967_SphI_F | AAAAGCATGCGCATAAATAGAGAGGACTTGTTAAAAAACCC |
| Sso0967_BamHI_R | AAAAGGATCCGCTTAATAAAGACGCGGCCAAAATAGG |
| aIF5A_N-His_NcoI_F | AAAACCATGGAACATCACCATCACCATCACAGCATAACGTACACGACCGTC |
| aIF5A_N-His_EagI_R | AAAACGGCCGTTACTTAACCCTAACTATTTTTCTC |
| aIF5A_C-His_NcoI_F | AAAACCATGGACAGCATAACGTACACGACCGTC |
| aIF5A_C-His_EagI_R | AAAACGGCCGTTAGTGATGGTGATGGTGATGCTTAACCCTAACTATTTTTCTCC |
| pMJ05_F | GGATGCTAAACAACTATTCAAACTG |
| pMJ05_R | GTTGTGTGGAATTGTGAGCGGATAA |

| **Strain** | **Description** | **Source / Reference** |
| --- | --- | --- |
|  |  |  |
| *E.coli* | DH5 | Invitrogen |
|  | DH5 (pETM11-N-His-aIF5A) | This work |
|  | ROSETTA (DE3) / pLysS | Novagen |
|  | ROSETTA (DE3) / pLysS (pETM11-N-His-aIF5A) | This work |
|  | TOP10 F1 | Invitrogen |
|  | TOP10 F1 (pQE-70-aDHS-C-His) | This work |
|  | BL21 (DE3) | Thermo scientific |
|  | BL21 (DE3) (pQE-70-aDHS-C-His) | This work |
|  |  |  |
| *S.solfataricus* | P2 | She et al. (2001) |
|  | PH1-16 | Schleper et al. (1992) |
|  | PH1-16 (pMJ05-ptf55α) | This work |
|  | PH1-16 (pMJ05-N-His-aIF5A) | This work |
|  | PH1-16 (pMJ05-aIF5A-C-His) | This work |

| **Plasmid** | **Resistance / Description** | **Source / Reference** |
| --- | --- | --- |
|  |  |  |
| pETM11 | Kanamycin | Provided by Dr. R.Spurio, University of Camerino (Italy) |
| pETM11-N-His-aIF5A | Kanamycin | This work |
| pQE-70 | Ampicillin | Quiagen |
| pQE-70-aDHS-C-His | Ampicillin | This work |
| pSVA11 | Ampicillin  Entry vector-tf55α constitutive promoter | Provided by Prof. S.V Albers, University of Freiburg (Germany) |
| pSVA11-N-His-aIF5A | Ampicillin  Entry vector-tf55α constitutive promoter | This work |
| pSVA11-aIF5A-C-His | Ampicillin  Entry vector-tf55α constitutive promoter | This work |
| pMJ05 | Ampicillin  Shuttle vector | Albers et al. (2006) |
| pMJ05-ptf55α | Ampicillin  Shuttle vector-tf55α constitutive promoter | This work |
| pMJ05-N-His-aIF5A | Ampicillin  Shuttle vector with tf55α constitutive promoter | This work |
| pMJ05-aIF5A-C-His | Ampicillin  Shuttle vector with tf55α constitutive promoter | This work |

**References**

She Q, Singh RK, Confalonieri F, Zivanovic Y, Allard G, Awayez MJ, Chan-Weiher CC, Clausen IG, Curtis BA, De Moors A, Erauso G, Fletcher C, Gordon PM, Heikamp-de Jong I, Jeffries AC, Kozera CJ, Medina N, Peng X, Thi-Ngoc HP, Redder P, Schenk ME, Theriault C, Tolstrup N, Charlebois RL, Doolittle WF, Duguet M, Gaasterland T, Garrett RA, Ragan MA, Sensen CW, Van der Oost J. (2001) The complete genome of the crenarchaeon *Sulfolobus solfataricus* P2. Proc. Natl. Acad. Sci. U.S.A. 98:7835–7840.

Schleper C, Kubo K, and Zillig W. (1992) The particle SSV1 from the extremely thermophilic archaeon *Sulfolobus* is a virus: demonstration of infectivity and of transfection with viral DNA. Proc Natl Acad Sci USA 89:7645–7649.

Albers SV, Jonuscheit M, Dinkelaker S, Urich T, Kletzin A, Tampe R, Driessen AJ, Schleper C. (2006) Production of recombinant and tagged proteins in the hyperthermophilic archaeon *Sulfolobus solfataricus*. Appl Environ Microbiol 72:102–111.
