# Supplementary figures and images for: Modification of translation factor aIF5A from Sulfolobus solfataricus
Source: Extremophiles. 2018 Jul 25;22(5):769–80. doi: 10.1007/s00792-018-1037-4 (PMC6105217; doi:10.1007/s00792-018-1037-4)

## Slide 1
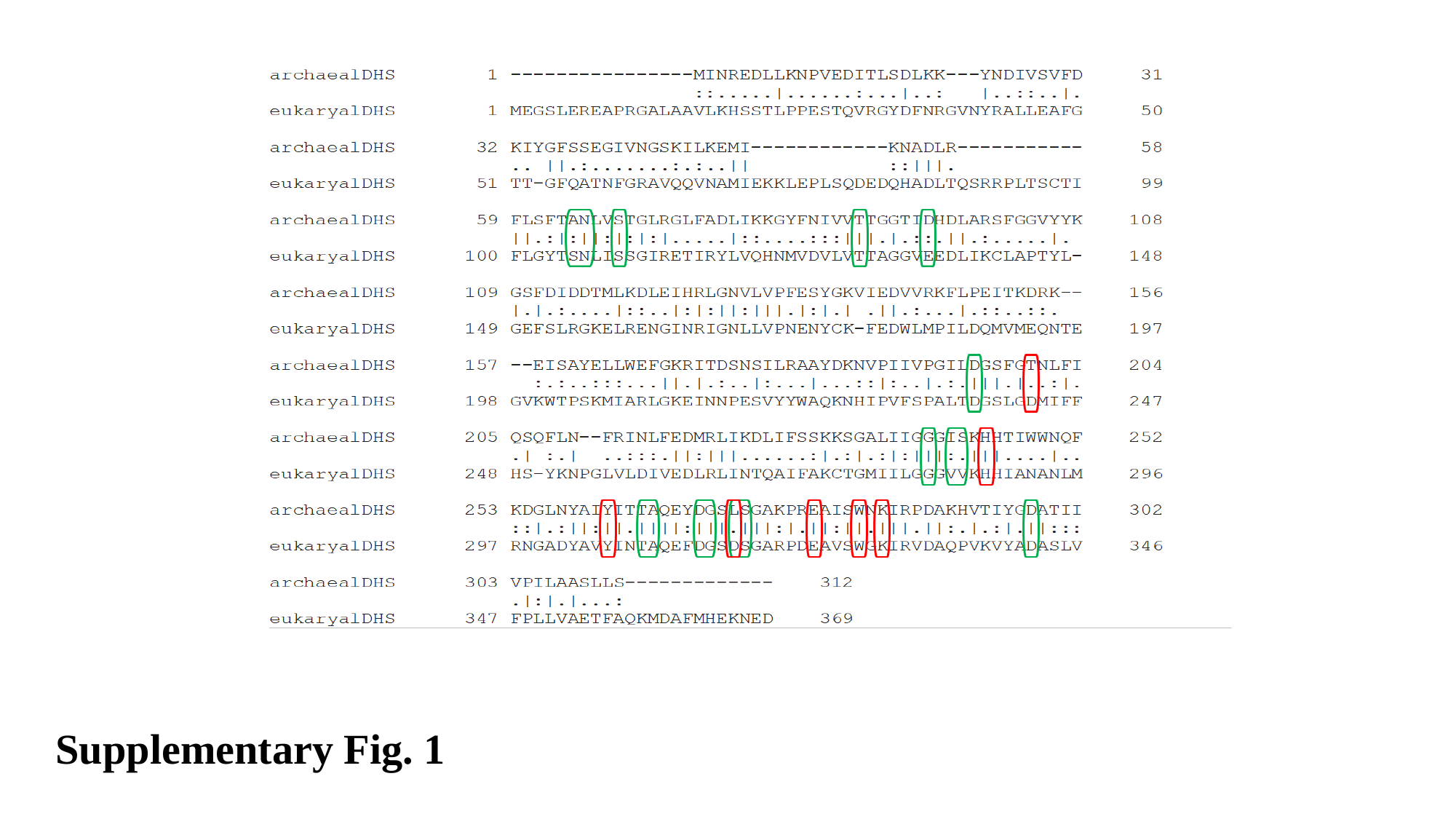

Supplementary Fig. 1

## Slide 2
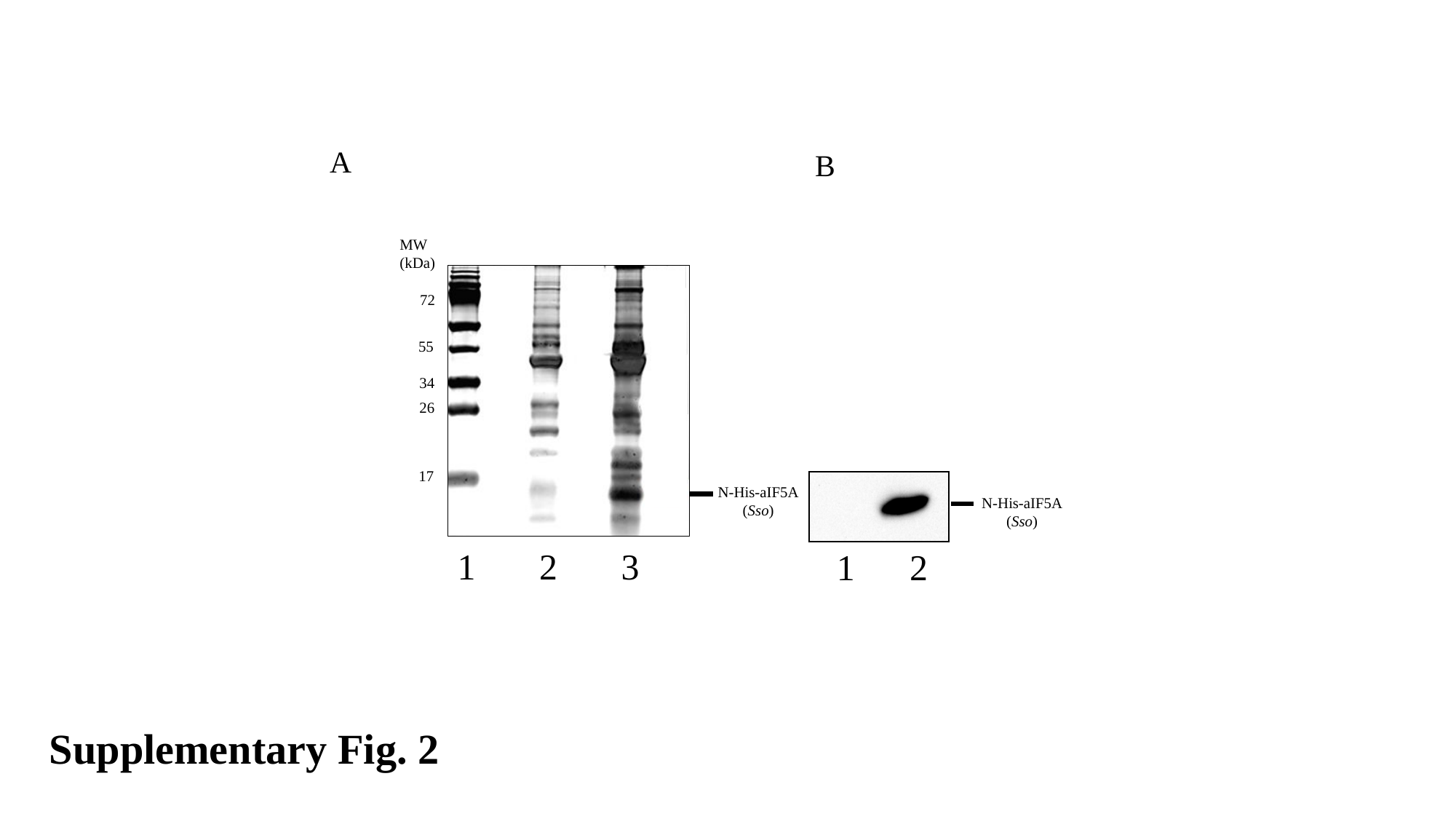

A
B
MW
(kDa)
72
55
34
26
17
N-His-aIF5A
(Sso)
N-His-aIF5A
(Sso)
1 2 3
1 2
Supplementary Fig. 2

Supplement: Supplementary file 2 — Supplementary material 2 (PPTX 385 kb) [file 792_2018_1037_MOESM2_ESM.pptx]
